# YouTube as a Source of Hidradenitis Suppurativa Patient Education: A Social Media Content Analysis

**DOI:** 10.7759/cureus.38047

**Published:** 2023-04-24

**Authors:** Daniela P Raime, Nada Ezaldein, Maynela Quiñones, Kingsley Chow, Kara Lukas, Harib H Ezaldein

**Affiliations:** 1 Dermatology, Miami Dermatology and Mohs Surgery, Miami, USA; 2 Dermatology, Bennett Surgery Center, Santa Monica, USA

**Keywords:** social media study, skin condition, dermatology surgery, dermatology, hidradenitis suppurativa

## Abstract

Hidradenitis suppurativa (HS) is a chronic and painful skin condition that is difficult to treat. Patients commonly navigate YouTube’s platform for insight into different treatment options; thus, we analyzed the content and quality of the top 100 HS videos to assess which treatment options were most favored. Our study indicated a growing number of informational videos on the platform over 10 years, with the majority of the content from the United States. Surgical videos had higher view counts than nonsurgical ones, even though the level of engagement measured by likes and comments was similar between the two. There were no differences in the presented tone between the two categories. Overall, YouTube videos have a moderate quality with no serious shortcomings based on a previously validated DISCERN instrument score. Healthcare professionals treating HS patients should continue to direct patients to evidenced-based sources of reliable information on their condition.

## Introduction

Hidradenitis suppurativa (HS) is a chronic and painful skin condition consisting of nodules and draining sinuses in intertriginous areas, possibly originating from follicular hyperkeratosis or rupture that leads to chronic perifollicular and periadnexal inflammation [1,2]. The skin condition is managed non-surgically with antibiotics, intralesional steroids, and/or systemic immunosuppression, while procedural treatments range from incision and drainage to deroofing, laser treatments, and surgical excision. As the inflammation diminishes, scarring, tunneling, and new sinuses can form underneath the skin, which can lead to additional flares or recurrences, even after procedural treatments [3,4]. Quality of life with HS is significantly impaired and worsens with the severity of the disease (i.e., number of affected areas, anogenital localization, and presence of nodules and fistulas) [5]. It is associated with many inflammatory comorbidities, in particular, obesity and being overweight, comprising 50-75% of the HS patient population [2].

Despite the morbidity of this condition, there is a general lack of HS awareness, which may explain why patients experience an average diagnostic delay of seven to eight years from initial symptoms to proper diagnosis [1]. Social platforms such as YouTube significantly expand the accessibility of health information for patients with chronic conditions via content that discusses personal experiences, the latest research, and medical, surgical, and alternative treatments [6].

The aim of our study is to characterize the quality of evidence depicted to viewers of the most popular YouTube videos regarding HS. We also seek to determine whether the content may favor alternative or homeopathic treatments compared to medical or surgical ones. Lastly, we seek to ascertain patterns of user engagement and feedback for content creators that may shift viewers' interest toward a specific treatment.

## Materials and methods

Study design

This is a cross-sectional study during which data was initially accessed and then reassessed at a future date. On March 27, 2021, the phrase “Hidradenitis Suppurativa” was searched on the platform YouTube. The search was sorted by video “relevance” in order to list the top 100 videos on this date. Only videos in English are included in the study. Uploaded videos in a foreign language, with or without English subtitles, or videos with time lengths of less than one minute are excluded. Promotional videos from independent content creators or product manufacturers to promote a product are also excluded.

Video characteristics

When the videos were initially accessed on March 27, 2021, several data points were collected for each video including the upload date, video length, number of views, total user-generated likes per video, total number of comments per video, total user-generated likes for the top comment per video, channel subscriber count, number of promotional advertisements, and DISCERN score. All data points were recorded and analyzed with descriptive statistics in Microsoft Excel®. When data were reassessed for methodological rigor, on July 27, 2022, the category “number of dislikes” was excluded due to YouTube’s removal of dislikes counts.

Content analysis

The content of the video was categorized by content category (e.g., education, quality of life, surgery, alternative medicine, etc.), research focus, treatment category (e.g., surgical or non-surgical), and tone of video (neutral, negative, or positive).

Personal experience videos

Video content discussing personal experience was further analyzed for the depicted individuals and topics of discussion: symptoms, medications, alternative medicine, and procedures.

Patient education videos and scoring system

We used the previously validated DISCERN score to evaluate the quality of the videos researched, originally developed to “enable information providers and patients to judge the quality of written information about treatment choices, facilitate the production of high-quality evidence-based consumer health information by setting standards, and provide a reference point for authors” [7]. This will allow us to compare the quality of treatment being provided regarding HS in the YouTube videos researched. The DISCERN score is rated on a scale of 1-5, including a list of questions created by the University of Oxford.

## Results

Initially, 100 videos (n=100) were analyzed for the research of HS, out of which only 38% of videos were surgical-based and 62% (the majority of the videos) were non-surgical-based. It was found that almost all the videos stayed relevant and in the top 100 at the second analysis time point. The list of videos gathered during the first search on March 27, 2021, was kept the same; however, the number of views, likes, comments, and subscribers was updated. Videos in our data were published from 2011 to 2021 with the highest number of videos published in 2019 and 2020: 23 (n=23) and 25 (n=25) videos, respectively (Table 1).

**Table 1:** A tabulation of key data points collected for the cohort over time

	2011	2012	2013	2014	2015	2016	2017	2018	2019	2020	2021	Total
Video Count, Total	1	2	1	2	1	6	17	12	23	25	10	100
Mean Length of Video (minutes)	5.72	9.34	3.48	3.96	2.67	10.99	9.97	8.98	11.55	10.38	11.73	10.24
Median Length of Video (minutes)	5.72	9.34	3.48	3.96	2.67	7.54	9.18	5.44	10.08	8.67	9.21	8.31
Mean View Count	973	64,339	45,053	83,379	22,960	454,220	212,070	151,587	216,891	37,375	11,780	145,546
Median View Count	973	64,339	45,053	83,379	22,960	54,377	69,423	10,583	31,651	11,939	5,752	18,794
Mean Number of Channel Subscribers	183	4,040	14,000	524,000	0	114,883	2,376,795	80,445	367609	371,397	80,937	616,797
Median Number of Channel Subscribers	183	4,040	14,000	524,000	0	161,00	32,200	2,270	74,800	34,400	24,350	32,200
Mean Number of Comments per Video	0	63	0	0.5	13	89.7	385.6	147.6	476.5	141.84	67.9	241.9
Median Number of Comments per Video	0	63	0	0.5	13	6.5	178	35.5	261	68	9	77
Mean Number of Ads per Video	0	0	0	0	0	1.17	0.35	0.92	0.69	0.92	0.6	0.69
Median Number of Ads per Video	0	0	0	0	0	1	0	0.5	0	0	0	0
Mean Number of Likes per Video	3	175.5	138	654	139	1005	2,437	1,419	5,606	751	429.5	2184.29
Median Number of Likes per Video	3	175.5	138	654	139	65	1200	245.5	600	282	148.5	333
Mean Number of Likes of Top Comment per Video	0	20.5	0	0	11	17.67	179.53	60.75	384.3	52.72	13.5	142.31
Median Number of Likes of Top Comment per Video	0	20.5	0	0	11	3	67	8	40	20	1	18
Mean DISCERN Score	1.37	2.59	2.31	3	2.75	2.39	2.84	2.71	2.53	2.14	2.76	2.52
Median DISCERN Score	1.37	2.59	2.31	3	2.75	2	3.06	2.56	2.53	1.94	2.5	2.44

The average length of videos was 10 minutes and 14 seconds. There was an average of 145,546 views from all the videos analyzed. The median total user-generated likes per video were 333. The median total number of comments per video was 241.9. The median total user-generated likes for the top comment per video were 18. The median channel subscriber count was 32,200. The average number of promotional advertisements per video was 0.69. The average DISCERN instrument score for all analyzed videos was 2.52.

Source of information

**Figure 1:**
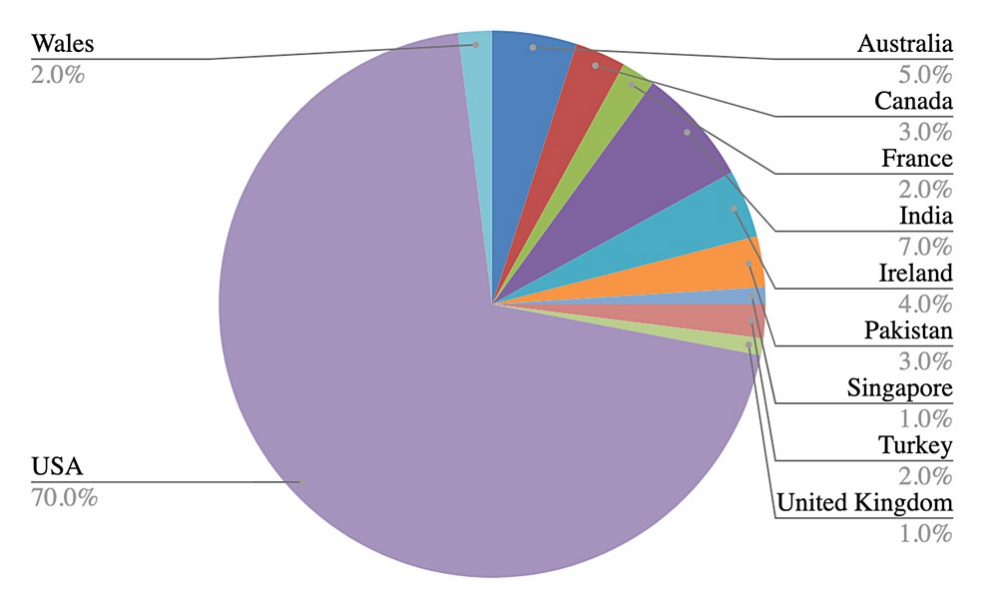
Video countries of origin The geographical distribution of videos in our study cohort.

Figure 1 compares video origin. Seventy percent (70%) of videos discussing HS were created in the United States compared to 30% created by other countries such as Australia, Canada, France, and India, among others. Videos made by individuals with financial gain had significantly more views than those provided by healthcare professionals and all other categories. The YouTube platform has created a bias on individuals with financial gain and surgical videos as the top surgical video having 2,531,383 views was created by a healthcare professional with a financial interest. A grand total of 14,554,648 views and 218,429 likes are counted across all videos in the study cohort. Twenty-eight (n=28) videos out of the 100 videos analyzed depicted personal sharing experiences. A grand total of 1,539,363 views and 23,748 likes accompanied this category. One YouTube account published five (n=5) videos in the top 100 and had a total of 1.26 million subscribers. Two non-surgical videos created by this account had 2,432,167 views with 96,000 likes and 125,236 views with 7,300 likes respectively, placing both videos in the top 20.

Thirty (n=30) videos were created by board-certified professionals and 70 (n=70) videos by non-board-certified professionals. There was more engagement on videos made by board-certified professionals with an average of 296,688 views compared to 80,771 of non-board-certified professionals (Table 2).

**Table 2:** A tabulation of key data points filtered by board-certified professionals

	Yes	No	Total
Video Count	30	70	100
Mean View Count	296,688	80,771	145,546
Median View Count	58,592	14,425	18,794
Mean Number of Likes per Video	1474.7	2488.4	2184.3
Median Number of Likes per Video	364	333	333
Mean Number of Comments per Video	182.6	267.3	241.9
Median Number of Comments per Video	41.5	84	77

Surgical vs non-surgical videos

**Figure 2:**
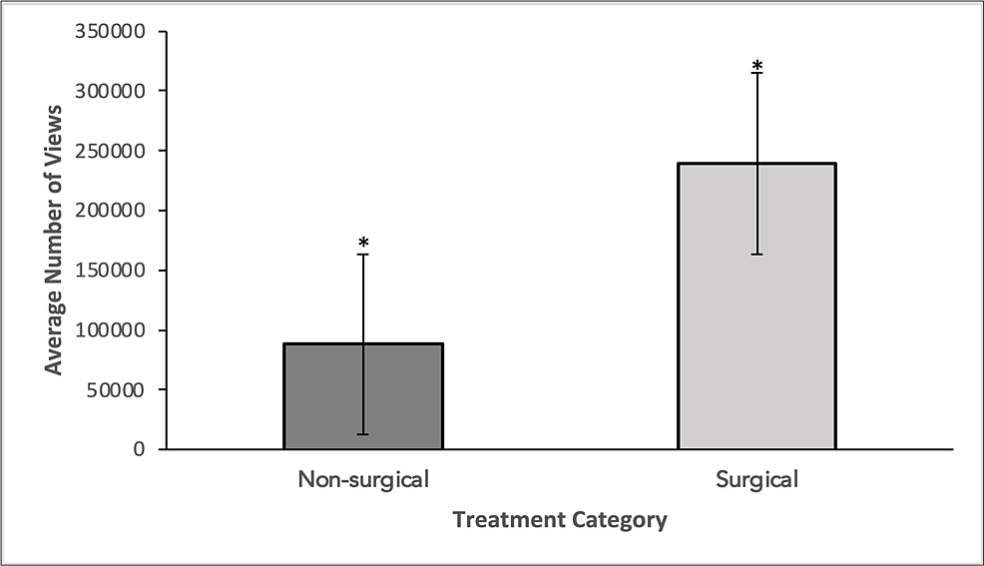
A comparison of the average number of views between non-surgical and surgical video categories Groups were compared using a two-tailed student’s t-test. A statistically significant difference was seen in the average number of views between non-surgical and surgical video categories (p-value = .044).

While the results showed that only 38% of the videos were surgical versus 62% non-surgical, other data showed the level of engagement from the public in these YouTube videos. Surgical videos had an average view count of 239,340 compared to 88,060 for non-surgical ones (Figure 2).

**Figure 3:**
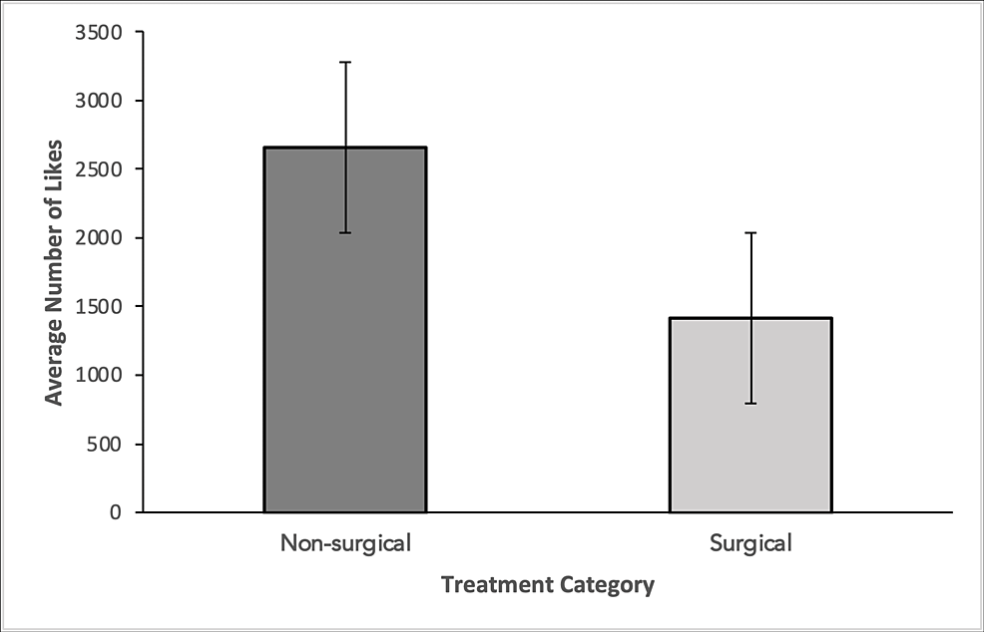
Average number of likes is depicted among non-surgical and surgical video categories Groups were compared using a two-tailed student’s t-test. No significant difference was observed between the two groups (p-value = .21).

This shows that people are watching more surgical treatment-related videos. In contrast, surgical videos had a 1,415 average number of likes compared to 2,655 for non-surgical videos (Figure 3).

**Figure 4:**
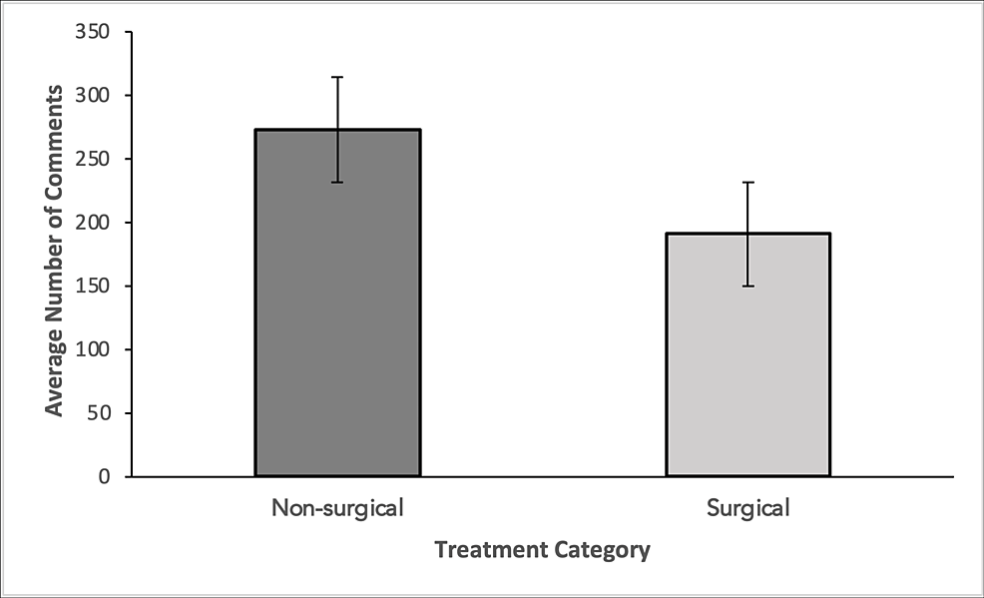
Average number of comments are compared between non-surgical and surgical video categories Groups were compared using a two-tailed student’s t-test. No significant difference was observed between the two groups (p-value = .26).

Similarly, surgical videos had an average number of comments of 190 versus 273 for non-surgical videos (Figure 4). This proves that non-surgical videos had more likes and comments than surgical videos which indicates a higher level of engagement with non-surgical videos.

Tone of video

All videos were categorized regarding the overall tone of the video which resulted in three different tones: negative, neutral, or positive (Table 3). Eighty-six (n=86) videos had a neutral tone, 53 (n=53) of the videos were non-surgical, and 33 (n=33) were surgical. In addition, there was an equal number of positive toned videos for both surgical and non-surgical with a total of eight (n=8) videos. Lastly, out of all the videos, six (n=6) were negatively toned, five (n=5) of which were non-surgical, and one (n=1) of which was surgical.

**Table 3:** Analysis of video tone, categorized by content into either surgical or non-surgical A chi-squared statistic was performed and did not yield statistical significance (p-value =.4375 and χ2 = 1.653).

	Positive	Neutral	Negative	Total
Surgical	4	53	5	62
Non-surgical	4	33	1	38
Total	8	86	6	100

Sixteen months later

There was a median increase of 14,500 subscribers from March 27, 2021, to July 27, 2022. Overall, there was an increase in engagement from all types of YouTube videos regarding HS. There was no change in the top 100 videos regarding HS on YouTube.

Patient education

Table 1 shows the median DISCERN score of 2.44. It also shows how DISCERN has fluctuated throughout the years since when the videos were made. Only one video had a DISCERN score of 5. This video was a patient-sharing experience video and had a positive overall tone.

Surgical alternative options

Topical treatments, such as homeopathic or prescription creams, botulinum toxin injections, and lasers, are considered alternative options to surgery for this study. Sixteen (16%) videos discussed alternative options to surgery including botulinum toxin, lasers, and topicals, while 18 (18%) discussed lifestyle modification, nine (9%) discussed topical treatments only, and nine (9%) discussed both alternative medicine plus lifestyle modifications.

## Discussion

Our study indicates a considerably growing and valuable amount of information on HS available to the public on YouTube™. While many videos are available for the condition of HS, we found that no new videos are being created or they are not showing enough quality to be in the 100 top videos searched. This was significant because when the data was analyzed 16 months later, the same videos appeared in the top 100 videos. With over 14 million views for the top 100 videos, we report a significant variability in the quality of information that exists, stemming from content creator identity, the accuracy of the information, commercial interests, or biases toward certain forms of medical treatment. Anecdotal videos are popular because they are easily relatable, but unfortunately, the poor quality of evidence in these videos enjoys a higher impact than higher-quality evidence-based content [8].

The variability and clinical accuracy of the content of such videos can be a source of concern due to their undue influence on patient care. A recent study on vaccine hesitancy concluded that significant misinformation exists and that negatively toned videos had a higher amount of sharing, likes, and views than positively toned ones [9]. Another study concluded that users could develop a watch history with misinformation that algorithmically recommends additional videos that also promote misinformation, a so-called “filter bubble effect” [8]. As the second largest social network in the world, YouTube is currently undertaking measures to curb healthcare misinformation through a special verification process aimed to bring high-quality health information to viewers [10]. This verification effort requires healthcare-focused channels to adhere to specific guidelines partially defined by health authorities such as the World Health Organization [11]. The effect of such efforts remains to be studied and may not be successful for videos in foreign languages but likely represents an avenue to improve the quality of information sourcing from the platform.

Our analysis indicated that although medical and surgical treatments for HS comprise the majority of views from the videos on YouTube, viewers seemed more engaged with those of complementary and alternative medicinal practices. The results showed that non-surgical videos had more likes and comments than surgical videos which indicates a higher level of engagement with non-surgical videos. Therefore, patients are open to surgical treatments but feel more comfortable with non-surgical treatment. Some of these findings are echoed in other studies, including one showing a negative correlation between scientific quality and viewer engagement. Some reasons for this are an abundance of anecdotal viewpoints and lack of expert consensus content, decreased health literacy of the viewership, commercialization of content creators, and that a growing number of users utilize comment sections of popular videos for social support [12]. HS is also a chronic skin condition that is difficult to treat and may wax and wane with conventional medical treatment. This may allow patients to experiment with and attribute some treatment efficacy to homeopathic or alternative medical treatments. Recent studies show that botulinum toxin and diode laser are widely used therapies when individuals fail topical creams or refrain from surgery. Six studies encompassing 31 patients showed that all patients tolerated treatment well with clinical improvement and clinical remission ranging around 6-12 months [13]. Patients reported improvement in hyperhidrosis but minimal change in HS disease severity.

Although surgery is not a cure for this disease, content related to surgical treatment attracted the most views. This could be motivated by a variety of reasons including the limited efficacy of topical or oral antibiotics leading patients to seek more drastic measures and perceived as permanent treatment options. Studies indicate that while more conservative medical management options are not curative, surgical intervention can have some curative effect through excision. However, there is still no complete cure, and relapse is frequently seen post-surgery [4]. In addition, the popularity of YouTube videos discussing surgery for HS could also be driven by preoperative anxiety, fear, and hesitancy to undergo surgery, and researching surgical outcomes can have a calming effect. Studies show that 73% of patients scheduled to undergo surgery have substantial preoperative anxiety [14].

A key drawback of our study includes sampling limitations to the top 100 videos in the English language. This lowers the likelihood of including videos featuring research or medical advice from healthcare professionals. We also did not consider the algorithmic video recommendations from YouTube after each video in our study. Therefore, a viewer's video experience may be different from the methodology used in our study. Although many of these suggested videos were frequently also in the search results generated from the protocol in our study, it is theoretically possible that the user experience and content exposure could be very different. Another drawback is the inability of knowing the audience, whether it is a healthcare provider, a patient, etc.

Our study indicated that there is an increasing awareness of HS, as individuals are more inclined to gain knowledge and understanding of the skin condition through online social platforms such as YouTube. Although board-certified healthcare professionals comprise a very small portion of the overall content creators, the platform is attempting to improve the quality of information through verification efforts for content creators. It is, thus, important for physicians to discuss the variability in the quality of information on YouTube with patients and actively refer them to online resources that accurately guide their decision-making and patient care.

## Conclusions

As the disease of HS becomes increasingly more common, it is important to understand the different types of treatments available to treat the condition. The YouTube platform gives access for patients to be able to explore the different options. The results of the data collected suggest that fewer surgical or medical treatments are recommended by content creators to the public. This suggests a growing role for healthcare providers to engage viewers on the YouTube platform to show the effectiveness of medical treatments toward HS.
